# Low-Speed Fault Diagnosis of Machinery Based on Improved Spectral Amplitude Modulation with Advanced Denoising Methods

**DOI:** 10.3390/s26154805

**Published:** 2026-07-28

**Authors:** Yukai Zhao, Yuncheng Guo, Yu Shang, Xiaojia Zu, Rongsheng Lin, Haihong Tang

**Affiliations:** 1 School of Marine Engineering Equipment, Zhejiang Ocean University, Zhoushan 316022, China; 2 Jurong Energy (Xinjiang) Co., Ltd., Urumqi 841603, China; 3 Jurong Petroleum and Natural Gas University-Enterprise Joint Laboratory, Zhejiang Ocean University, Zhoushan 316022, China

**Keywords:** fast spectral coherence, spectral amplitude modulation, low-speed bearing fault diagnosis, cyclostationary analysis, squared enhanced envelope spectra

## Abstract

**Highlights:**

(i) **FSC is applied to noise suppression.** In FSC, the fault feature frequency is the cyclic frequency when a rolling bearing fault occurs. This process can effectively reduce irrelevant interference components in the modified signal and separate valid information related to the fault.

(ii) **SEESs is applied to feature enhancement.** In SEESs, fault characteristic information of bearings buried in background noise and miscellaneous interferences can be captured. Periodic enhancement of frequency-domain information further concentrates and amplifies faint energy components, enabling the identification of incipient weak fault signatures.

**What are the main findings?**

- The proposed FSC-SAM method effectively overcomes the limitations of traditional SAM, achieving prominent noise suppression and weak fault feature enhancement for low-speed bearing signals contaminated by heavy interference.
- Both simulated signals with different SNRs and experimental data of bearing inner and outer race faults at 60 RPM verify that FSC-SAM can stably extract complete fault characteristic frequencies and their harmonic components, which conventional methods fail to identify clearly.

**What are the implications of the main findings?**

- This hybrid signal processing approach provides a reliable technical solution for incipient fault diagnosis of offshore wind turbine bearings operating under low-speed and harsh marine working conditions.
- The method helps reduce unplanned equipment shutdowns, cut high offshore maintenance costs, and improve the overall operational stability and economic benefits of wind turbine units.

**Abstract:**

Heavy background noise inevitably masks the weak fault characteristics of low-speed bearings in offshore wind turbines, severely degrading the fault detection accuracy of conventional spectral amplitude modulation. To address this challenging issue, a novel hybrid diagnosis method integrating fast spectral coherence and spectral amplitude modulation is proposed for low-speed bearing fault detection. Firstly, spectral amplitude modulation is implemented on raw vibration signals to construct modified signal components. Subsequently, the fast spectral coherence algorithm is employed to eliminate complex noise interference and reconstruct high-quality enhanced envelope spectra. Further squaring transformation and amplitude normalization operations are conducted to effectively amplify incipient and weak fault signatures buried in noisy signals. Multiple visual analysis strategies are also adopted to intuitively present the fault diagnosis results. The effectiveness and superiority of the proposed method are comprehensively validated by simulated signals with varying signal-to-noise ratios and experimental data of bearing inner and outer race faults under a low rotating speed of 60 RPM. The results demonstrate that the proposed hybrid method significantly remedies the inherent limitations of traditional diagnosis methods, providing a robust and reliable technical solution for incipient fault monitoring and health assessment of low-speed bearings operating in harsh offshore wind field environments.

## 1. Introduction

Rolling bearings are the core and critical components of offshore wind turbines [1,2]. Affected by the harsh offshore operating environment, the bearings of offshore wind turbines are particularly susceptible to failure, which directly leads to shutdowns, exacerbates economic losses, and increases potential safety risks [3]. Therefore, it has great practical significance to conduct timely and accurate fault diagnosis for the bearings of offshore wind turbines to ensure their reliable and stable operation, especially under low speed [4].

The technique of extracting features from signals using signal processing methods is a crucial research direction in the field of bearing fault diagnosis [5]. The core reason is that the mutual collision between rolling elements and the defective surface leads to the generation of repetitive impacts in the vibration signal when a bearing defect occurs [6]. Thus, signal processing techniques can excavate abundant fault information from the vibration signal and extract valuable fault features [7]. Consequently, a series of signal processing methods have been continuously emerging and developing [8], among which the most common one is envelope analysis [9]. Envelope analysis first performs band-pass filtering on the original vibration signal within a specific high-frequency band and subsequently conducts amplitude demodulation on the filtered signal to obtain the envelope signal, whose frequency spectrum contains the required fault information [10,11].

However, the primary challenge of envelope analysis lies in the considerable difficulty in selecting the correct resonant frequency band. To address the aforementioned issues, researchers proposed Autogram to determine the optimal demodulation frequency band while reducing irrelevant interference by calculating the kurtosis of the unbiased autocorrelation of the squared envelope of the demodulated signal [12]. Furthermore, a harmonic spectral kurtosis is proposed to suppress impulsive interference, which ensures that the filtered frequency band contains more fault information [13]. Considering the time-varying nature of bearing vibration signals, Hou [14] developed a sparse time-frequency analysis method to reveal the sparsity of fault signatures and improve the readability of time-frequency representations under variable-speed operating conditions. A second-order synchrosqueezing transform integrated with reassignment technology is proposed for feature characterization of variable-speed vibration signals, which enhances the energy concentration and resolution of time-frequency ridges [15]. From the perspective of order tracking, researchers [16,17] have tried to extract modulation features from nonstationary signals to sharpen fault impulses and construct holographic Hilbert order spectra. However, the aforementioned methods still have the following two issues: **(i) optimal demodulation frequency band.** At low speeds, the impact signals are severely submerged by noise interference, which renders the aforementioned methods unable to accurately locate the optimal demodulation frequency band; **(ii) the simultaneous occurrence of multiple faults.** When multiple fault features are distributed across different frequency bands, the aforementioned methods can usually only detect a single fault while neglecting others. This limitation becomes even more pronounced, especially in the case of low-speed conditions where the energy impact of faults is weak, thus resulting in incomplete fault diagnosis.

To overcome the aforementioned challenges, researchers proposed a novel nonlinear filtering algorithm that is entirely different from the aforementioned methods, namely spectral amplitude modulation (SAM) [18]. Especially, it possesses advantages for resolving the spectral blurring of main bearing signals under variable-speed conditions [19]. From the perspective of interference suppression, a novel adaptive residual spectral amplitude modulation (ARSAM) is proposed, which achieves nonlinear separation of fault features while removing interference components [20]. Furthermore, a researcher [21] proposed the Stockwell transform spectral amplitude modulation (STSAM), which employs the Stockwell transform to obtain amplitude and phase information with time-frequency characteristics, thereby reducing irrelevant interference and highlighting fault features [22]. An adaptive residual spectral amplitude modulation method is proposed to improve the interference suppression capacity for random impulses and harmonics, which concentrates on amplifying fault signatures within fault resonance frequency bands [23]. In terms of feature enhancement, adaptive spectral amplitude modulation is adopted to mitigate spectral overlap and strengthen fault characteristic frequencies as well as their harmonics [24]. However, at low speeds, the impulse signal energy of bearing faults is weak and easily masked by strong interference, making it impossible to accurately identify the weak fault features of low-speed bearings.

To address the aforementioned issues, a low-speed bearing fault diagnosis method based on fast spectral coherence and spectral amplitude modulation (FSC-SAM) is proposed from the perspectives of noise suppression and feature amplification. Firstly, SAM is applied to treat raw signals with different magnitude orders (MOs) to enhance the fault features in the signal and obtain the modified signal. Then, fast spectral coherence (FSC) is performed on the modified signal to further reduce irrelevant interference components, resulting in a series of enhanced envelope spectra (EESs). Moreover, the squared enhanced envelope spectra (SEESs) are applied to enhance feature information through squared operation in EESs. Finally, the experimental results are displayed through a three-dimensional plot, a two-dimensional plot, and the maximum squared enhanced envelope spectrum (MSEES). The main contributions of this paper are as follows.

(i) **FSC is applied to noise suppression.** In FSC, the fault feature frequency is the cyclic frequency when a rolling bearing fault occurs. This process can effectively reduce irrelevant interference components in the modified signal and separate valid information related to the fault.
(ii) **SEESs is applied to feature enhancement.** In SEESs, fault characteristic information of bearings buried in background noise and miscellaneous interferences can be captured. Periodic enhancement of frequency-domain information further concentrates and amplifies faint energy components, enabling the identification of incipient weak fault signatures.
(iii) **FSC-SAM is applied to a low-speed bearing.** The proposed method can effectively extract the fault feature from a low-speed bearing signal. The effective detection of weak faults in low-speed bearings helps reduce unplanned shutdowns of wind turbines.

## 2. Theory

### 2.1. Fast Spectral Coherence

**Figure 1:**
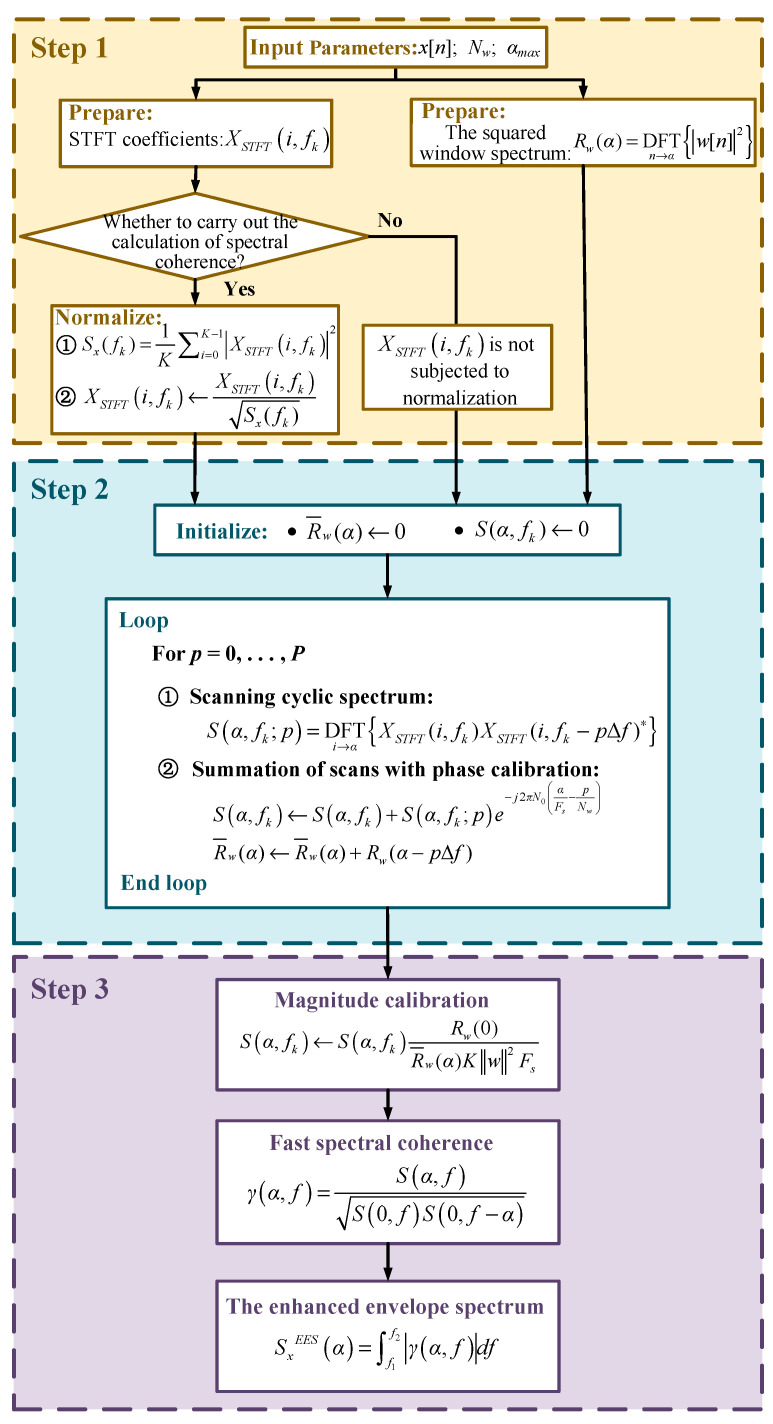
The algorithm block diagram of the FSC.

Spectral correlation (SC) is an important tool for analyzing cyclostationary signals, as it can extract specific cyclostationary components hidden in interference [25,26,27]. However, since SC is defined as the two-dimensional Fourier transform of the instantaneous autocorrelation function, this definition relies on ideal signals with infinite length [28]. In practical applications, signals are all finite-length noisy signals, making the theoretical value of SC unachievable directly [29]. Therefore, the theoretical quantity of SC must be approximated using estimators. The averaged cyclic periodogram (ACP) [30], the cyclic modulation spectrum (CMS) [31], and FSC [32] have been proposed as estimators of SC. The FSC is selected in this paper due to its higher computational efficiency and superior performance [33]. The algorithm block diagram of the FSC is shown in Figure 1 and the steps of the FSC are as follows.

**Set input parameters and prepare intermediate variables.** The input parameters include the raw vibration signal x(t), the window length $N_{w}$ in the Short-Time Fourier Transform (STFT), and the maximum cyclic frequency $\alpha_{max}$. The STFT coefficients of the raw vibration signal are denoted as

(1) $$\begin{array}{c} X_{STFT}\left(i,f_{k}\right)=\sum_{m=0}^{N_{w}-1}x[iR+m]w[m]e^{-j2\pi m\frac{f_{k}}{F_{s}}}\\ t_{n}=\frac{n}{F_{s}},\;f_{k}=k\Delta f=k\frac{F_{s}}{N_{w}} \end{array}$$

where *i* denotes the time index; $f_{k}$ represents discrete frequencies, $k=0,\cdots ,N_{w}-1$; R denotes the block shift in STFT; the value of R determines the value of the maximum cyclic frequency $\alpha_{max}$; $w\left[m\right]$ denotes the data window (function of time index m); $F_{s}$ denotes the sampling frequency; $t_{n}$ denotes the time instants; n=0,⋯,L−1, L is the signal length.

**Compute the scanning spectral correlation.** The scanning spectral correlation is given as follows

(2) $$\begin{array}{l} S_{x}\left(\alpha ,f_{k};p\right)=\frac{1}{K{\left\|w\right\|}^{2}F_{s}}\underset{i\to \alpha }{\mathrm{DFT}}\left\{X_{STFT}\left(i,f_{k}\right)X_{STFT}{\left(i,f_{k-p}\right)}^{*}\right\}e^{-j2\pi N_{0}\left(\frac{\alpha }{F_{s}}-\frac{p}{N_{w}}\right)}\\ {\left\|w\right\|}^{2}=\sum_{n=0}^{N_{w}-1}{\left|w[n]\right|}^{2} \end{array}$$

where DFT denotes the Discrete Fourier Transform; α is the cyclic frequency; p denotes the index of STFT frequency closest to a given cyclic frequency α; $N_{0}$ denotes the central time index of the window.

**Magnitude calibration.** The equation given below is proposed for magnitude calibration

(3) $$S\left(\alpha ,f\right)=\frac{\sum_{p=0}^{P}S_{x}\left(\alpha ,f;p\right)}{\sum_{p=0}^{P}R_{w}\left(\alpha -p\Delta f\right)}R_{w}(0)$$

where $R_{w}(•)$ represents the kernel function; f is the spectral frequency. Then, the FSC is computed as

(4) $$\gamma \left(\alpha ,f\right)=\frac{S\left(\alpha ,f\right)}{\sqrt{S\left(0,f\right)S\left(0,f-\alpha \right)}}$$

EES is obtained by integrating the magnitude of the FSC over frequencies [34].

(5) $${S_{x}}^{EES}\left(\alpha \right)=\int_{f_{1}}^{f_{2}}\left|\gamma \left(\alpha ,f\right)\right|df$$

where $\left[f_{1},f_{2}\right]$ is a given frequency band.

### 2.2. Spectral Amplitude Modulation

**Figure 2:**
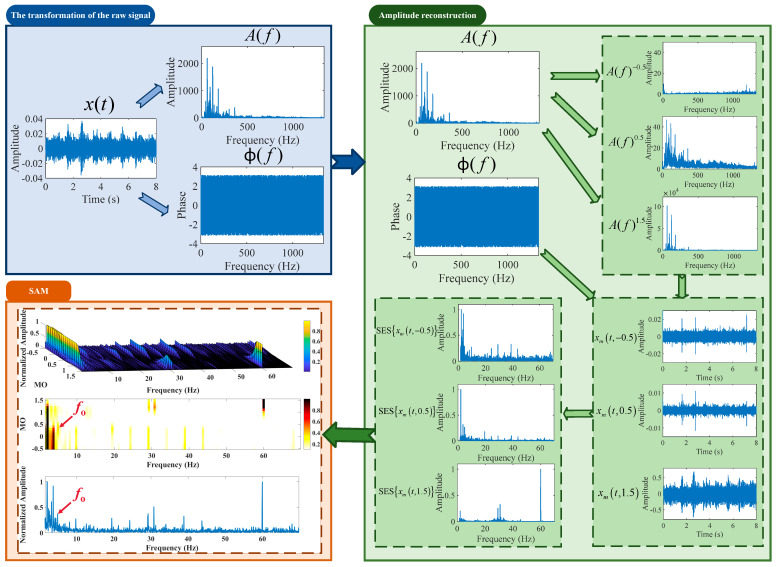
The flow block diagram of SAM.

SAM is equivalent to a nonlinear filtering process, enabling the separation of signals from different sources. Furthermore, it features simple computation, high efficiency, and no requirement for preset parameters. The flow block diagram of SAM is shown in Figure 2. The raw signal x(t) is defined as the input to SAM and then the decomposed signal is obtained as follows:

(6) $$X(f)=FT\left\{x(t)\right\}=A(f)e^{j\phi (f)}$$

where j is the imaginary unit and $FT\left\{⦁\right\}$ refers to the Fourier transform; A(f) is the magnitude, ϕ(f) is the phase.

The key step of SAM is to sequentially assign different magnitude orders (MOs) to the magnitude A(f) for obtaining $A{\left(f\right)}^{\mathrm{MO}}$; next combine it with the actual phase ϕ(f) of the raw signal; and then the modified signal is obtained via the inverse Fourier transform (IFT), thereby completing signal reconstruction:

(7) $$x_{m}(t,\mathrm{MO})=\mathrm{IFT}\left\{A{\left(f\right)}^{\mathrm{MO}}e^{j\phi (f)}\right\}$$

where $x_{m}\left(t,\mathrm{MO}\right)$ is the modified signal. This process is equivalent to a nonlinear filtering process, in which signals from different sources are separated. In Equation (7), MO = 1 is equivalent to the original amplitude spectrum with no feature enhancement; MO > 1 large-amplitude components (fault impact harmonics) are exponentially amplified, while small-amplitude random noise is suppressed to highlight fault signatures; 0 ≤ MO ≤ 1, the dominance of higher-amplitude frequency components gradually weakens relative to lower-amplitude ones as MO decreases; MO < 0, the amplitude effect is completely reversed: the amplitude of previously higher-amplitude frequency components becomes negligible, while the influence of previously lower-amplitude ones is enhanced. In this research, the recommended range for MO is from −0.5 to 1.5, with a step size of 0.1.

Finally, the modified signal $x_{m}\left(t,\mathrm{MO}\right)$ is used to derive the squared envelope spectrum (SES) via the Hilbert transform and Fourier Transform (FT):

(8) $$\begin{array}{l} \mathrm{SES}\left\{x_{m}(t,\mathrm{MO})\right\}=\left|\mathrm{FT}\left\{{\left|\Gamma \left\{x_{m}(t,\mathrm{MO})\right\}\right|}^{2}\right\}\right|\\ \Gamma \left\{x_{m}(t,\mathrm{MO})\right\}=x_{m}(t,\mathrm{MO})+j*\mathrm{Hilbert}\left\{x_{m}(t,\mathrm{MO})\right\} \end{array}$$

where $\mathrm{Hilbert}\left\{\cdot \right\}$ refers to the Hilbert transform.

The normalization of SES is crucial for balancing results across different MO values, as it ensures the clarity and comparability of the outcomes. The results are visualized via a three-dimensional plot and a two-dimensional plot. The maximum squared envelope spectrum (MSES) displays the results obtained along the MO axis, where the *x* and *y* axes represent the modulation frequency and normalized amplitude, respectively.

## 3. The Methodology of FSC-SAM

**Figure 3:**
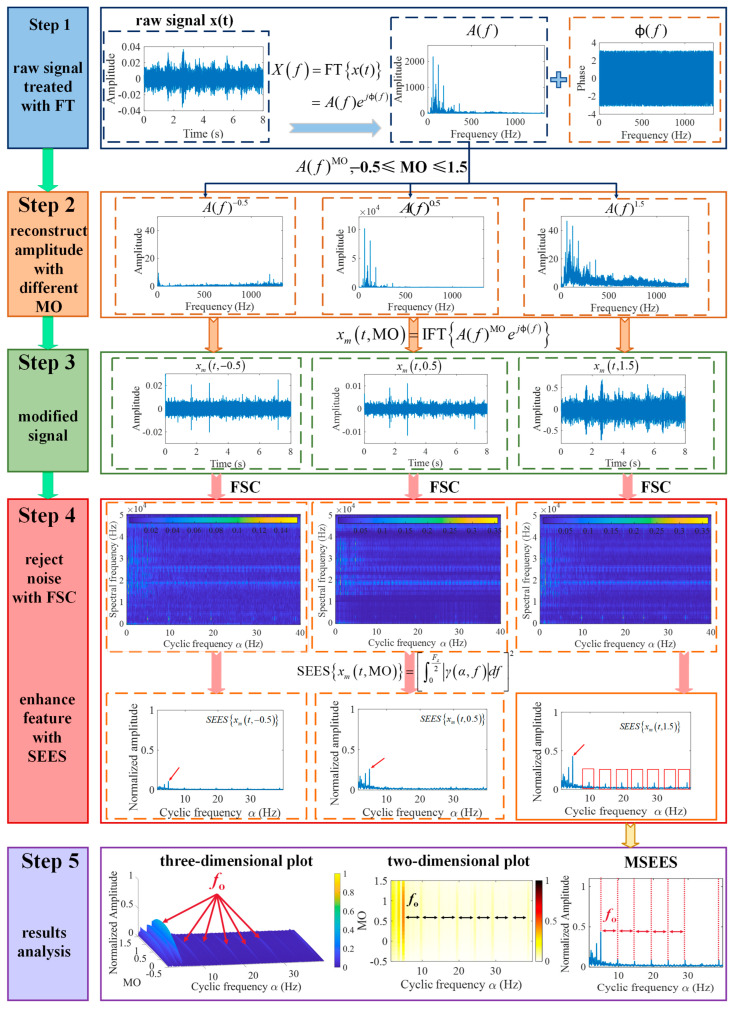
The flowchart of FSC-SAM.

In the extraction of bearing fault features, the following drawbacks need to be addressed when applying the traditional SAM to raw vibration signals: (i) although SAM assigns different weights to the amplitude of the raw signal to achieve the separation of signals from different sources, the modified signal still contains a large number of irrelevant interference components; (ii) at low speeds, the energy of fault impulse signals is relatively weak and prone to being masked, making it impossible to accurately identify fault features. A novel method named FSC-SAM is proposed to address the aforementioned issues. The implementation and description of each step are provided in detail below and the flowchart of FSC-SAM is illustrated in Figure 3.

### 3.1. FSC Analysis for the Modified Signals

The raw signal *x*(*t*) is first subjected to FT to obtain the magnitude A(f) and phase ϕ(f) according to Equation (6). Then, different MOs (−0.5 ≤ MO ≤ 1.5) are assigned to the magnitude A(f) to obtain the modified magnitude $A{\left(f\right)}^{\mathrm{MO}}$. Subsequently, the modified magnitude $A{\left(f\right)}^{\mathrm{MO}}$ is combined with the raw phase ϕ(f) to obtain the modified signal $x_{m}\left(t,\mathrm{MO}\right)$ according to Equation (7). Different weights are assigned to different frequency components, which may result in a nonlinear filtering process, thereby enabling the separation of signals from different sources.

However, since this method applies amplitude weighting across the entire frequency domain, the modified signal under each MO value contains a large number of interference components, thereby reducing the effectiveness of fault feature identification. To address the aforementioned issue, FSC is performed on a series of modified signals $x_{m}\left(t,\mathrm{MO}\right)$ according to Equations (1)−(4).

**Figure 4:**
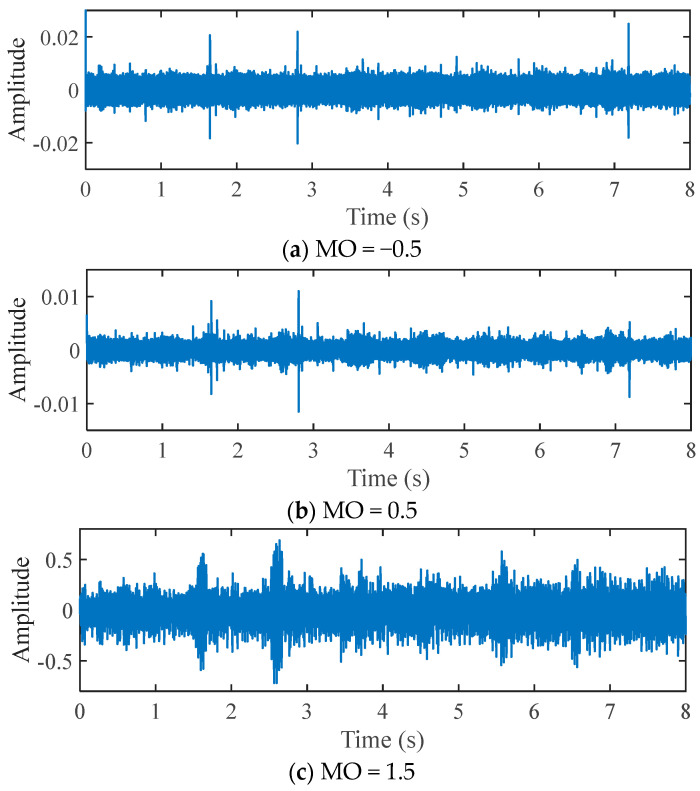
The modified signals.

**Figure 5:**
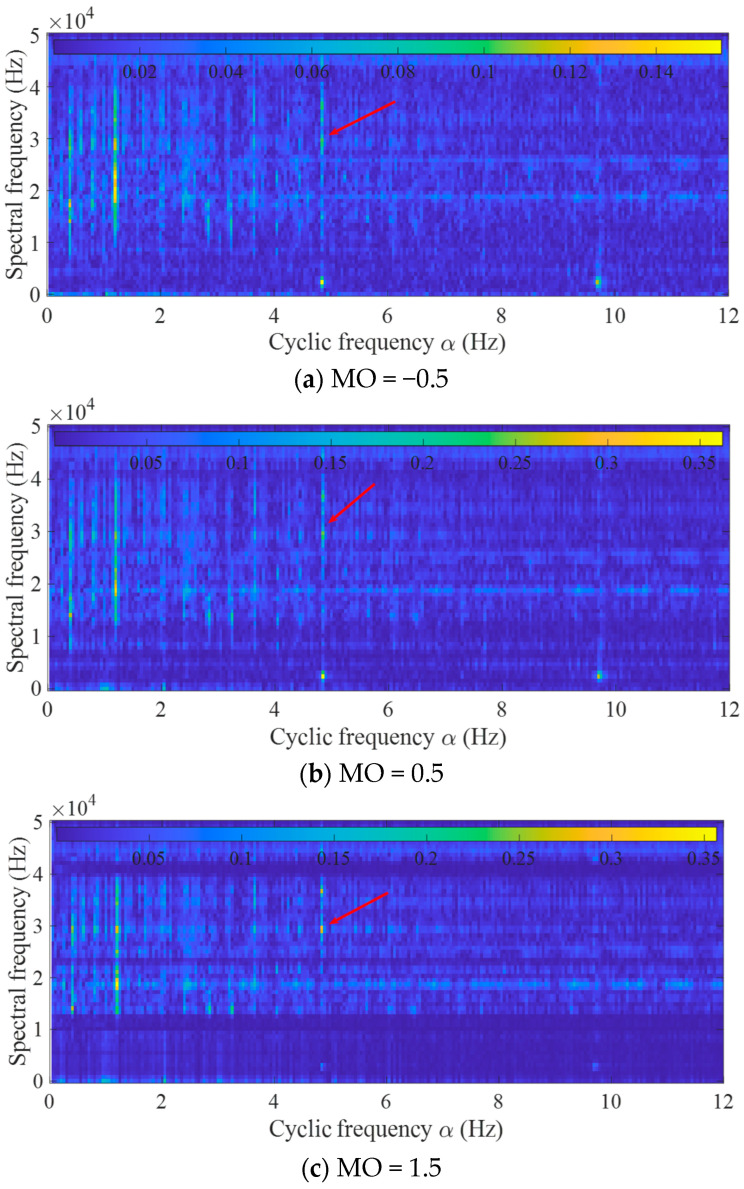
The FSC of the modified signals.

As shown in Figure 4 and Figure 5, examples are displayed associated with MO = −0.5, MO = 0.5, and MO = 1.5 for illustrative purposes when $N_{w}=27$ in this paper. In the FSC map, the brightness of a pixel represents the amplitude of cyclic modulation at a specific cyclic frequency and spectral frequency. When a bearing develops a fault, it generates a cyclostationary signal. The amplitude of the pixel associated with the corresponding cyclic frequency ($\alpha =f_{fault}$) and spectral frequency increases. The amplitude and number of harmonics associated with the cyclic frequency related to this fault can be used as indicators to evaluate the severity of the fault. Furthermore, FSC can suppress uncorrelated noise and highlight the modulation components induced by faults.

### 3.2. FSC-SAM Based on SEES

In bearing fault diagnosis, envelope spectrum analysis serves as a mainstream diagnostic technique for extracting periodic impulse characteristics. Nevertheless, there are two issues to be considered in low-speed bearing fault diagnosis. Firstly, the **(i) difficulty of extracting weak fault impulses.** Its reliability degrades sharply under low-speed operating conditions, where weak fault impulses are easily submerged by heavy background noise and complex structural interferences. Secondly, **(ii) selection of frequency bands in EES.** The selection of frequency bands generally relies on expert knowledge and field experience, which is why we adopt squared enhanced envelope spectra (SEES) to replace EES. To tackle this practical problem, this paper develops an improved FSC-SAM framework based on SEES. Benefiting from the joint advantages of fast spectral coherence and spectral amplitude modulation, SEES delivers remarkable noise reduction performance and greatly amplifies faint fault signatures, which facilitates accurate extraction of bearing fault characteristic frequencies and their corresponding harmonic components in harsh industrial scenarios.

SEES is expressed as

(9) $$\mathrm{SEES}\left\{x_{m}(t,\mathrm{MO})\right\}={\left[\int_{0}^{F_{s}/2}\left|\gamma (\alpha ,f)\right|df\right]}^{2}$$

where $F_{s}$ denotes the sampling frequency. SEESs are normalized as follows

(10) $$\mathrm{NSEES}\left\{x_{m}\left(t,\mathrm{MO}\right)\right\}=\frac{\mathrm{SEES}\left\{x_{m}\left(t,\mathrm{MO}\right)\right\}}{\max\left(\mathrm{SEES}\left\{x_{m}\left(t,\mathrm{MO}\right)\right\}\right)}$$

**Figure 6:**
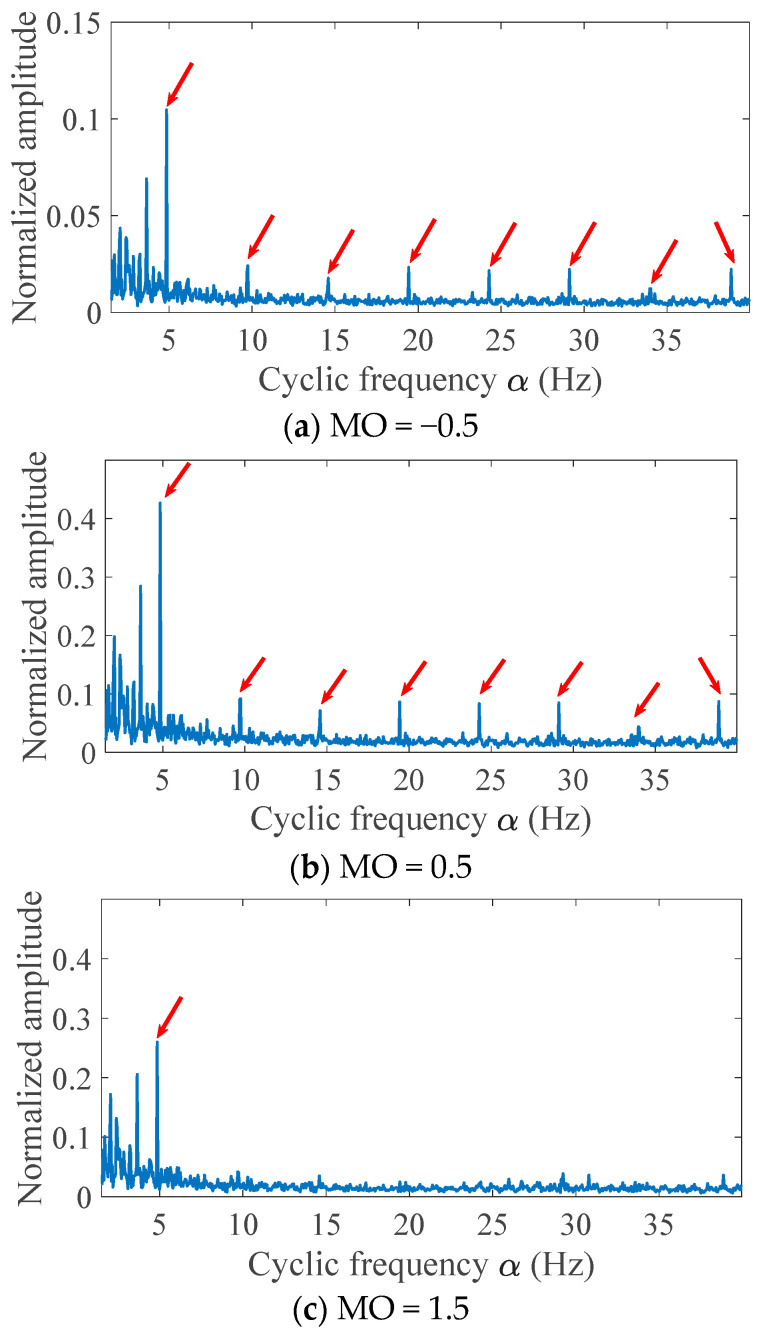
The normalized SEESs.

And the experimental results with NSEESs are shown in Figure 6. The frequency spectrum with different MOs to demonstrate the effectiveness of NSEESs.

## 4. Simulated Signal Analysis

In practical operating conditions, especially for the bearings of offshore wind turbines operating at low speeds, the raw signals, in addition to background noise and fault impact signals, may also contain low-frequency and high-frequency interference components, such as vibrations from supporting structures, shafts, and rotors, as well as vibrations from gears and other machine components. Therefore, a simulated vibration signal is generated to verify the effectiveness of the proposed method, as shown in Equation (11).

(11) $$\begin{array}{l} x(t)=m(t)+s(t)+n(t)\\ m(t)=Ae^{-2\pi \zeta f_{n}t^{\prime }}\sin\left(2\pi f_{n}\sqrt{1-\zeta^{2}}t^{\prime }\right)\\ s(t)=M\sin(2\pi f_{1}t+\beta )+M\sin(2\pi f_{2}t+\beta ) \end{array}$$

where m(t) represents the repetitive impulse signal caused by the outer race fault of the bearing; s(t) simulates the low-frequency and high-frequency interference components under actual conditions; n(t) denotes Gaussian white noise. The sampling frequency $f_{s}=20\;kHz$, the natural frequency $f_{n}=3\;kHz$, the amplitude A=5, the damping coefficient ζ=0.1, the fault feature frequency is 25 Hz, the amplitude M=3.5, $f_{1}=30\;Hz$, $f_{2}=850\;Hz$, β=40π.

**Figure 7:**
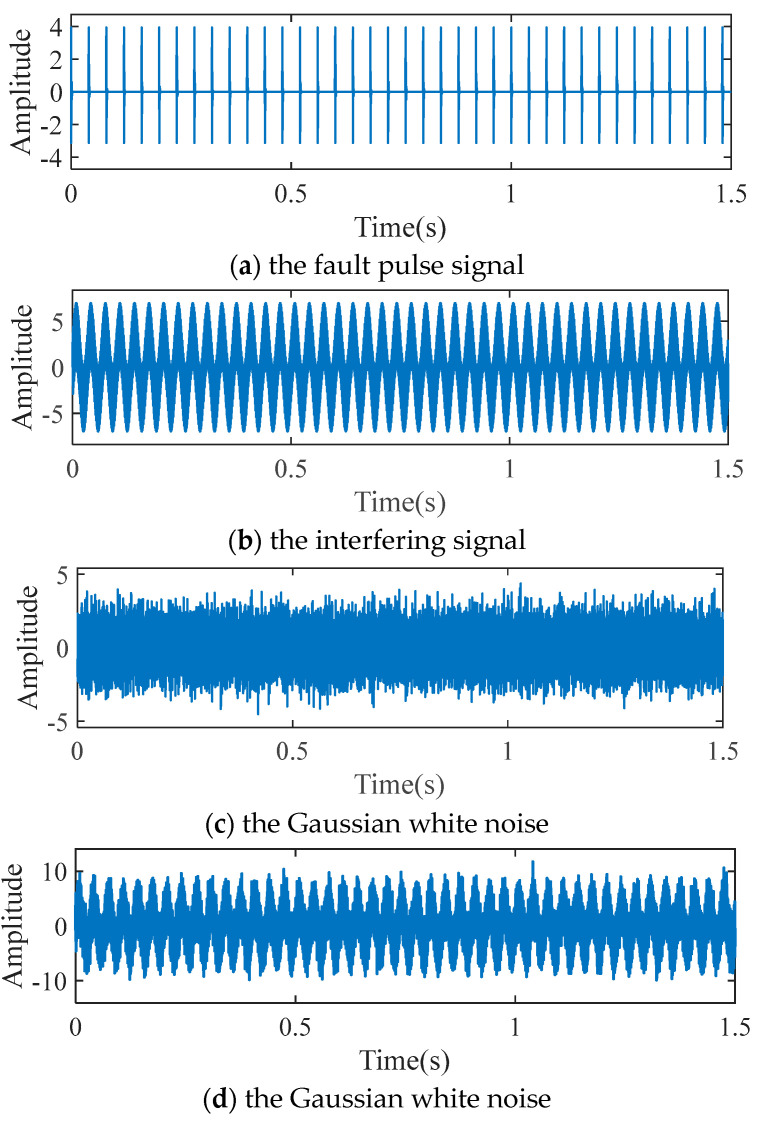

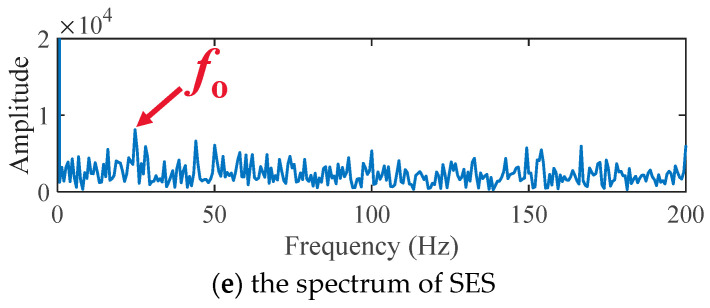
The results of simulated signal.

As shown in Figure 7, it presents the respective components, time domain, and envelope spectrum of the simulated signal with an SNR of −12 dB. As shown in Figure 7e, the fault pulse is completely masked by noise. In the envelope spectrum, only the fault feature frequency *f_o_* is weakly identifiable.

**Figure 8:**
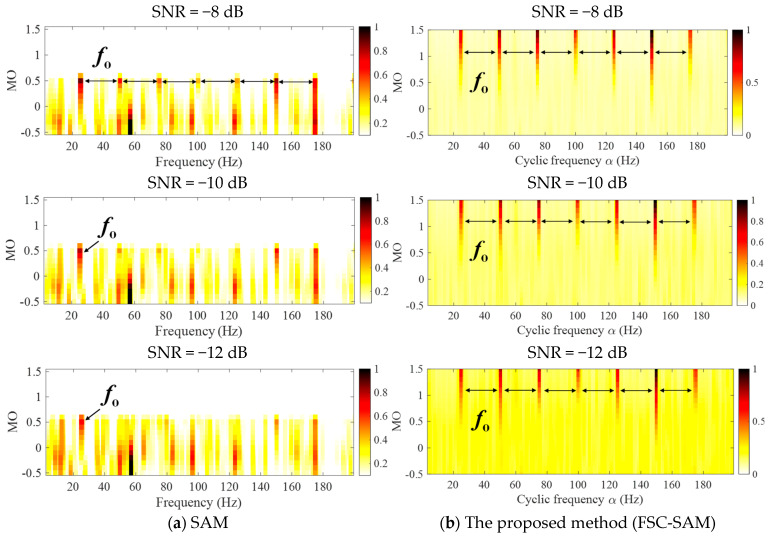
Analysis results of two-dimensional plots for SAM and FSC-SAM under different SNRs.

**Figure 9:**
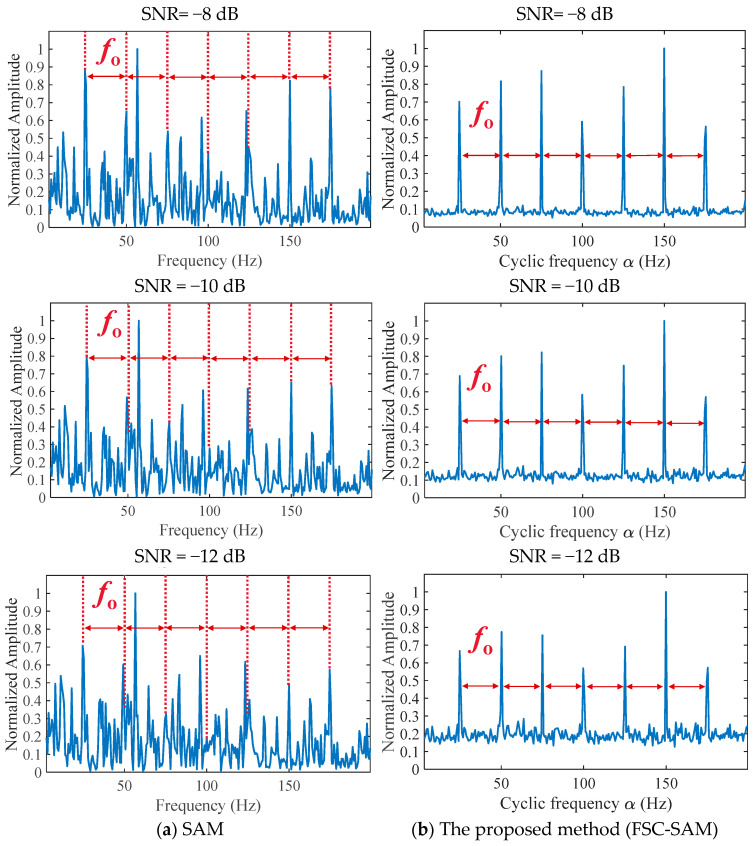
Spectrum for SAM and FSC-SAM under different SNRs.

As shown in Figure 8a, with the decrease in the SNR, the fault feature frequency *f_o_* and its harmonic frequencies can be observed to become increasingly indistinct. As shown in Figure 9a, with the increase in the SNR, the dominant position of fault features increases to a certain extent. However, due to the influence of interfering signals, the fault feature frequency *f_o_* and its harmonic frequencies still cannot be clearly extracted. Subsequently, the FSC-SAM method was applied to analyze the same signals. As shown in Figure 8b and Figure 9b, the fault feature frequency *f_o_* and its harmonics can be clearly detected under the three different SNRs. Therefore, through comparison, it can be concluded that the FSC-SAM exhibits more significant advantages in extracting fault features under the three different SNRs.

## 5. Experimental Analysis

### 5.1. Experimental Equipment

**Figure 10:**
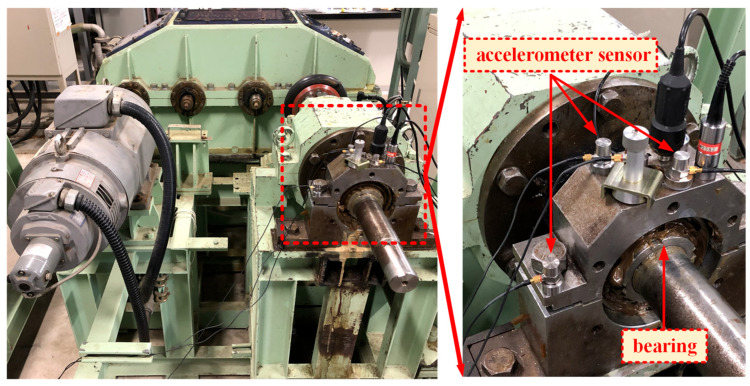
The experimental platform.

To verify the effectiveness of the proposed method in the fault diagnosis of bearings in offshore wind turbines, a single fault type analysis is performed using the experimental platform illustrated in Figure 10. For sensors, they are the accelerometer sensors (PCB MA352A60, PCB Piezotronics Inc., New York, NY, USA) that were fixed in the vertical direction of the bearing. The raw signals were transformed with a sensor signal conditioner (PCB ICP Model 480C02, PCB Piezotronics Inc., New York, NY, USA) and then those signals were saved with a signal recorder (Scope Coder DL750, Yokogawa Co. Ltd., Tokyo, Japan). The sampling frequency is 100 kHz, and the sampling time is 20 s. For the bearing, the types of outer and inner are N312 and NU312, respectively. The parameters of both bearings are shown in Table 1.

(12) $$\begin{array}{l} f_{o}=\frac{N_{b}f_{r}}{2}\left(1-\frac{D_{b}}{D_{c}}cosa\right)\\ f_{i}=\frac{N_{b}f_{r}}{2}\left(1+\frac{D_{b}}{D_{c}}cosa\right) \end{array}$$

where is the number of rollers $N_{b}=12$, roller diameter $D_{b}=18\;mm$, the bearing pitch diameter $D_{c}=90\;mm$, contact angle a=0, rotating frequency $f_{r}=60\;rpm/60\;s$.

**Figure 11:**
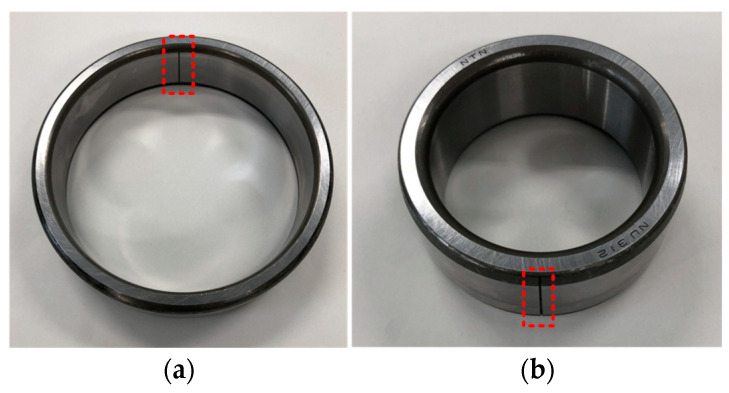
(**a**) Bearing outer fault; (**b**) bearing inner fault.

**Figure 12:**
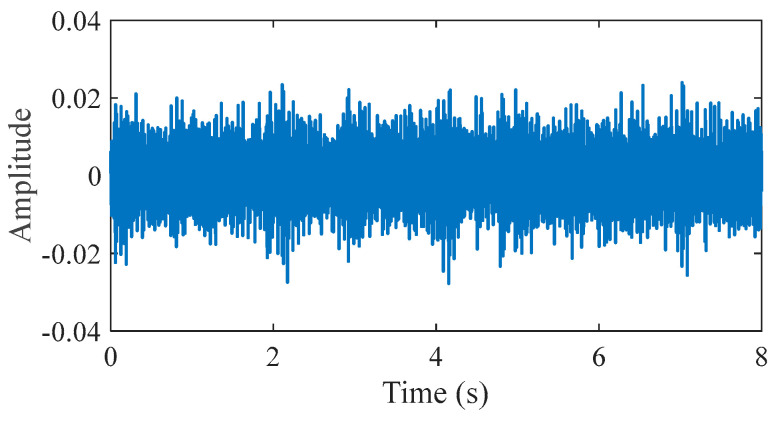
Bearing signal under normal conditions at 60 RPM.

The faulty bearing is shown in Figure 11. The time-domain waveforms of the bearing signals at 60 RPM under normal conditions are presented in Figure 12. The bearing parameters are listed in Table 2.

### 5.2. Experimental Results

#### 5.2.1. Outer Fault

**Figure 13:**
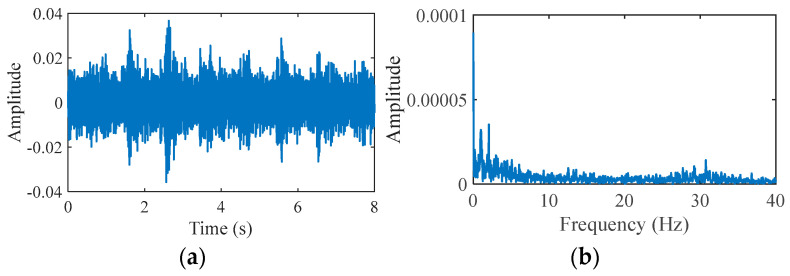
(**a**) The waveform of the raw vibration signal (Outer); (**b**) its SES.

The time-domain waveform and envelope spectrum of the raw vibration signal are shown in Figure 13. As shown in Figure 13a, the obvious periodic impacts in the time-domain waveform cannot be detected. As shown in Figure 13b, neither the fault feature frequency nor its harmonics can be found in the corresponding SES. Therefore, further analysis of the raw signal is necessary.

**Figure 14:**
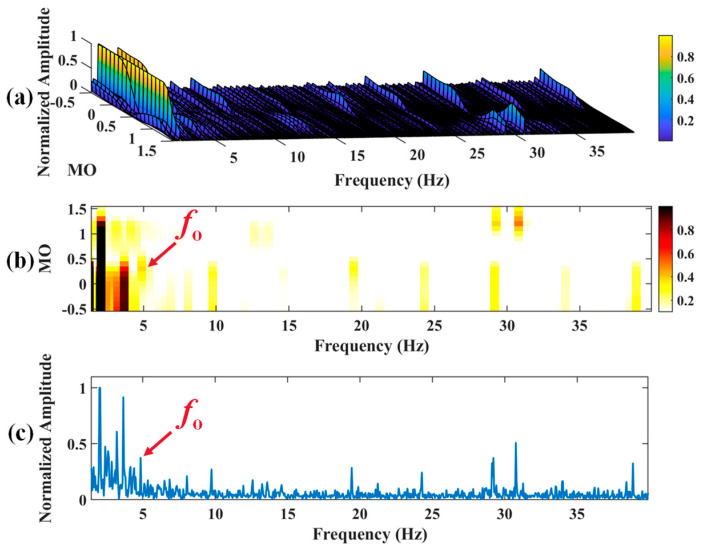
The result of SAM (Outer): (**a**) the three-dimensional plot; (**b**) the two-dimensional plot; (**c**) MSES.

As shown in Figure 14, the raw signal is analyzed using the SAM method. Figure 14b shows that the outer race fault feature frequency *f_o_* can only be weakly detected when 0.3 ≤ MO ≤ 0.5. The outer race fault feature frequency *f_o_* is also very weak in MSES. Therefore, the fault feature extraction capability of the traditional SAM is not prominent.

**Figure 15:**
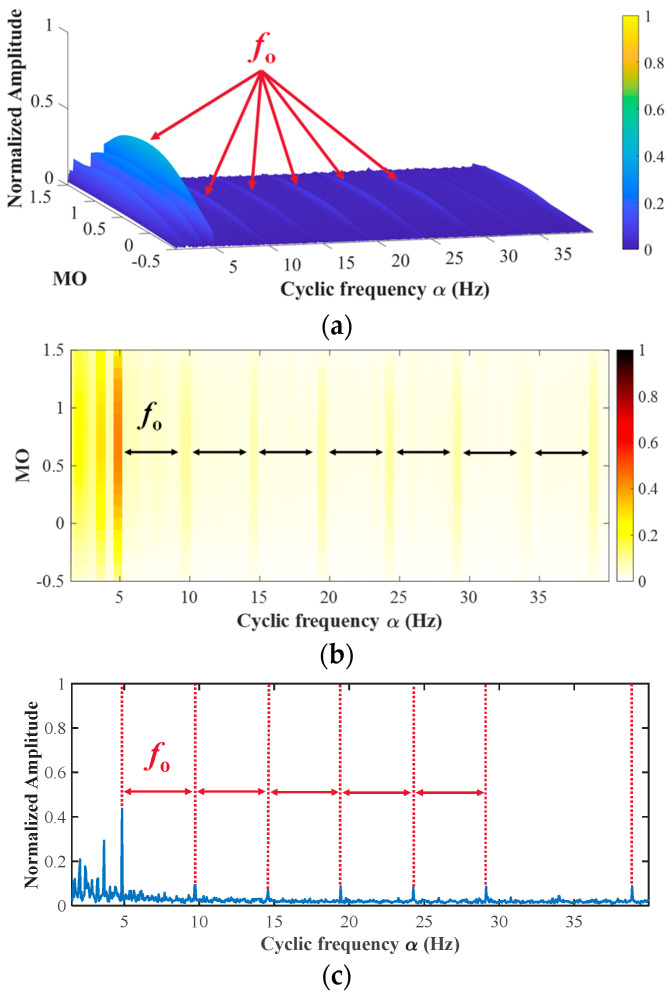
The result of FSC-SAM (Outer): (**a**) the three-dimensional plot; (**b**) the two-dimensional plot; (**c**) MSEES.

As shown in Figure 15, it presents the results of processing the raw signal using the FSC-SAM method. The maximum cyclic frequency *α_max_* is set to 40 Hz. As shown in Figure 15a, the outer race fault feature frequency *f_o_* and its harmonics are clearly identifiable in the three-dimensional plot. As shown in Figure 15b, the outer race fault feature frequency *f_o_* and its harmonics 2 *f_o_*, 3 *f_o_*, 4 *f_o_*, 5 *f_o_*, 6 *f_o_*, and 8 *f_o_* are relatively prominent when 0 ≤ MO ≤ 1. Only the outer race fault feature frequency *f_o_* is prominent when −0.5 ≤ MO ≤ 0 and 1 ≤ MO ≤ 1.5. In MSEES, the outer ring fault feature frequency *f_o_* and its harmonics 2 *f_o_*, 3 *f_o_*, 4 *f_o_*, 5 *f_o_*, 6 *f_o_*, and 8 *f_o_* can also be clearly observed.

Moreover, there are four metrics to evaluate signal quality including peak-to-peak value, kurtosis, skewness and crest factor. Those parameters are calculated with equations in [35], and the information is described in Table 3. Although SAM is outstanding in the large-scale converters field, the increase in fault signal parameter characteristic index is more resilient using FSC-SAM. The processing time of the entire algorithm flow is 2869 s.

#### 5.2.2. Inner Fault

**Figure 16:**
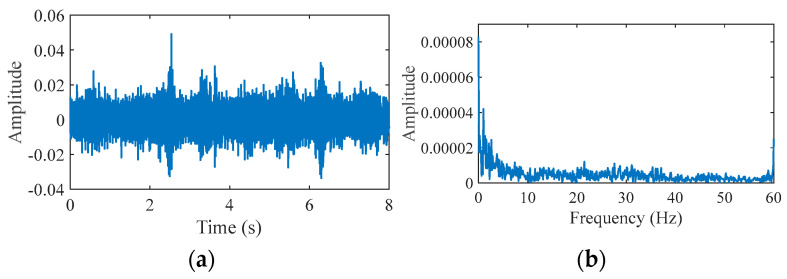
(**a**) The waveform of the raw vibration signal (Inner); (**b**) its SES.

The time-domain waveform and envelope spectrum of the raw vibration signal are shown in Figure 16. Since obvious fault information cannot be obtained from the time-domain waveform and the SES, further analysis of the raw signal is required.

**Figure 17:**
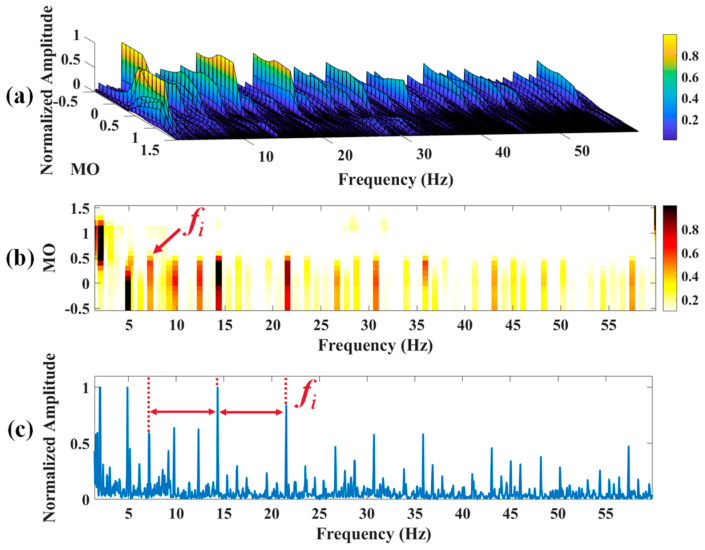
The result of SAM (Inner): (**a**) the three-dimensional plot; (**b**) the two-dimensional plot; (**c**) MSES.

**Figure 18:**
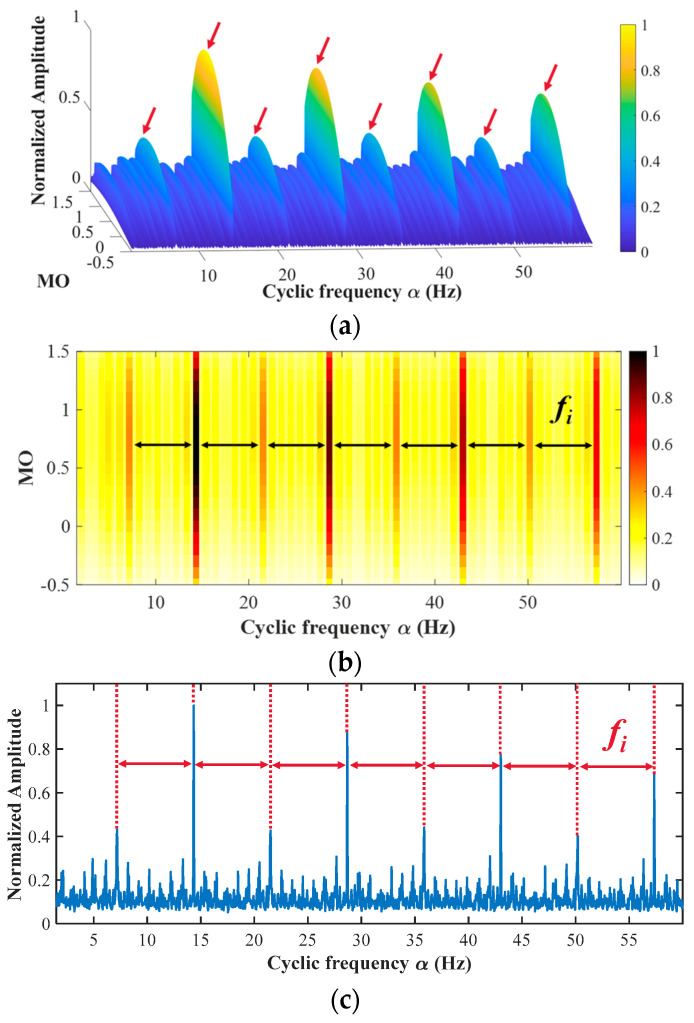
The result of FSC-SAM (Outer): (**a**) the three-dimensional plot; (**b**) the two-dimensional plot; (**c**) MSEES.

First, the raw vibration signal is analyzed using the SAM method. As illustrated in Figure 17b,c, the inner race fault feature frequency *f_i_* cannot be clearly observed due to noise interference. Therefore, it is necessary to perform feature extraction with the proposed method (FSC-SAM). As shown in Figure 18, it presents the results of processing the raw vibration signal using the FSC-SAM method. The maximum cyclic frequency *α_max_* is set to 60 Hz.

As shown in Figure 18a, the inner race fault feature frequency *f_i_* and its harmonics can be clearly detected. As depicted in Figure 18b, the inner race fault feature frequency *f_i_* and its harmonics can be clearly detected when 0 ≤ MO ≤ 1.4. In MSEES, the inner race fault feature frequency *f_i_* and its harmonics can also be clearly identified. As shown in Figure 18b, the inner race fault feature frequency *f_i_* and its harmonics 2 *f_i_*, 3 *f_i_*, 4 *f_i_*, 5 *f_i_*, 6 *f_i_*, and 8 *f_i_* are relatively prominent when 0 ≤ MO ≤ 1. Only the outer race fault feature frequency *f_i_* is prominent when −0.5 ≤ MO ≤ 0 and 1 ≤ MO ≤ 1.4. In MSEES, the inner ring fault feature frequency *fi* and its harmonics 2*f_i_*, 3*f_i_*, 4*f_i_*, 5*f_i_*, 6*f_i_*, and 8*f_i_* can also be clearly observed. Through comparison with the SAM method, it is found that the proposed method can effectively extract the inner race fault features.

Moreover, there are four metrics to evaluate signal quality including peak-to-peak value, kurtosis, skewness and crest factor, as shown in Table 4. SAM demonstrates reliable diagnostic capability; nevertheless, the proposed FSC-SAM approach enables a more substantial enhancement of fault feature characteristic indexes. The processing time of the entire algorithm flow is 3124 s.

### 5.3. Analysis and Discussion

The comparative results between the FSC-SAM method and SAM clearly demonstrate the superior performance of FSC-SAM in low-speed bearing fault diagnosis under strong noise interference. In simulated signal tests with a signal-to-noise ratio (SNR) ranging from −8 dB to −12 dB, SAM gradually lost the capability to distinguish fault characteristic frequencies and their harmonic components as noise intensity increased, owing to its limited denoising ability against irrelevant interference retained after amplitude weighting. By contrast, the introduction of FSC effectively excavates cyclostationary fault components hidden in noisy signals, suppresses uncorrelated background noise and structural vibration interference, and retains valid fault-related modulation information. The squaring operation on enhanced envelope spectra further amplifies weak impulse features induced by bearing defects, which fundamentally addresses the issue that low-speed fault impulses are easily submerged by noise. Meanwhile, the normalization processing of squared enhanced envelope spectra unifies the amplitude scale under different magnitude order (MO) values, ensuring the intuitiveness and comparability of two-dimensional and three-dimensional visual results and the maximum squared enhanced envelope spectrum, which facilitates quantitative and qualitative identification of fault characteristics.

Experimental validation on actual offshore wind turbine bearing test rigs under 60 RPM low-speed operating conditions further verifies the practical applicability of FSC-SAM. For both outer race and inner race bearing faults, raw vibration signals and conventional envelope spectra failed to capture obvious periodic impact features and fault frequencies due to weak fault energy and complex on-site interference. Traditional SAM could only faintly detect fault characteristics within a narrow MO interval, and the fault harmonic components were almost indistinguishable, indicating its deficiency in extracting weak low-speed fault features. In comparison, FSC-SAM stably extracted complete fault characteristic frequencies and multiple harmonics across a wide range of MO values. The three-dimensional and two-dimensional spectra presented distinct and concentrated fault feature peaks, and the maximum squared enhanced envelope spectrum clearly displayed the amplitude distribution of each harmonic component. This phenomenon proves that the combination of FSC and SAM achieves complementary advantages: SAM realizes nonlinear separation of signal components via amplitude weighting, while FSC further purifies the modified signals from the perspective of cyclostationary analysis. The proposed method not only overcomes the defects of conventional demodulation analysis methods in selecting optimal frequency bands under low-speed conditions but also improves the detection accuracy for single-point bearing faults, providing reliable technical support for early fault warning and condition monitoring of low-speed rotating machinery in harsh marine service environments.

## 6. Conclusions

To address the problem that fault signals of offshore wind turbine bearings are often contaminated with noise interference under harsh offshore operating conditions, especially at low speeds, which makes it difficult for traditional SAM to identify fault features, a low-speed bearing fault diagnosis method based on FSC and SAM is proposed. First, the proposed method assigns different weights to the amplitude of the raw vibration signal to enhance the fault features, thereby obtaining the modified signal. Second, it performs FSC analysis on the modified signal to further suppress irrelevant interference components, resulting in a series of EESs. The squared operation is applied to the EESs to further highlight the fault features, yielding the SEESs. Finally, the SEESs are normalized, and the fault diagnosis results are visualized through a three-dimensional plot, a two-dimensional plot, and the MSEES, thereby strengthening the weak fault features at low speeds. The experimental results show that the FSC-SAM method can effectively identify fault information and successfully diagnose low-speed bearing faults. Compared with other techniques, its advantages are further demonstrated.

## 7. Shortcomings and Future Research

Although the proposed FSC-SAM delivers satisfying weak fault feature enhancement and noise suppression for low-speed offshore wind turbine bearing diagnosis, it has inherent limitations originating from the core mechanism of fast spectral coherence (FSC). As a frequency-domain linear correlation metric, FSC only quantifies linear signal coupling and cannot reflect nonlinear dynamic correlations at all. Real offshore operation involves complex mechanical excitations, stochastic marine noise, and fault evolution, which generate strong nonlinear coupling between fault impulses and environmental interferences. When fault and noise signals follow nonlinear driving relations, FSC coefficients may approach zero despite their high intrinsic correlation, resulting in degraded feature extraction and failed capture of nonlinear fault dynamics. Restricted to signals with linear frequency-domain correlations, this method fails to handle working conditions dominated by nonlinear signal coupling. Future work will introduce nonlinear signal processing theories to offset FSC’s linear constraint. A hybrid linear-nonlinear feature enhancement framework will be developed to adapt to the complicated nonlinear vibrations of offshore wind turbine bearings, boosting the generalization and practicability of incipient fault diagnosis in real engineering.

## Figures and Tables

**Table 1:** The detailed information of the bearing.

Speed	Inner Diameter	Outer Diameter	Roller Diameter
60 RPM	87.5 mm	113 mm	18 mm
**Number of Rollers**	**Bearing Pitch Diameter**	**Contact Angle**	**Rotating Frequency**
12	90 mm	0	1 Hz

**Table 2:** The detailed information of fault features frequency.

Bearing	Width × Depth	Frequency
Outer	0.6 mm × 0.3 mm	*f_o_* = 4.8 Hz
Inner	0.6 mm × 0.3 mm	*f_i_* = 7.2 Hz

**Table 3:** Comparisons with dimensionless parameters between different methods (Outer).

Signal	Dimension Parameters	Dimensionless Parameters		
	Peak-to-Peak	Kurtosis	Skewness	Crest
**Raw Signal**	9.46	11.03	3.38	10.53
**SAM**	10.65	9.29	10.49	16.28
**FSC-SAM**	**14.67**	**22.65**	**15.71**	**18.69**

**Table 4:** Comparisons with dimensionless parameters between different methods (Inner).

Signal	Dimension Parameters	Dimensionless Parameters		
	Peak-to-Peak	Kurtosis	Skewness	Crest
**Raw Signal**	26.65	2.57	1.04	6.61
**SAM**	31.47	3.81	2.79	4.18
**FSC-SAM**	**35.67**	**5.93**	**4.55**	**10.49**

## Data Availability

The data that has been used is confidential.

## Abbreviations

SAM	spectral amplitude modulation
ARSAM	adaptive residual spectral amplitude modulation
STSAM	Stockwell transform spectral amplitude modulation
FSC-SAM	fast spectral coherence and spectral amplitude modulation
MO	magnitude order
FSC	fast spectral coherence
EESs	enhanced envelope spectra
SEESs	squared enhanced envelope spectra
MSEES	maximum squared enhanced envelope spectrum
SC	Spectral correlation
ACP	averaged cyclic periodogram
CMS	cyclic modulation spectrum
STFT	Short-Time Fourier Transform
DFT	Discrete Fourier Transform
FT	Fourier Transform
